# Primary Osteoporosis in Young Adults: Genetic Basis and Identification of Novel Variants in Causal Genes

**DOI:** 10.1002/jbm4.10020

**Published:** 2017-11-06

**Authors:** Corinne Collet, Agnès Ostertag, Manon Ricquebourg, Marine Delecourt, Giulia Tueur, Bertrand Isidor, Pascale Guillot, Elise Schaefer, Rose‐Marie Javier, Thomas Funck‐Brentano, Philippe Orcel, Jean‐Louis Laplanche, Martine Cohen‐Solal

**Affiliations:** ^1^ Department of Biochemistry and Genetics Hospital Lariboisiere Paris France; ^2^ INSERM U1132 University Paris‐Diderot Paris France; ^3^ Department of Rheumatology Hospital Lariboisiere Paris France; ^4^ Genetic Medical Department Centre Hospitalier Universitaire (CHU) de Nantes Nantes France; ^5^ Genetic Medical Department les Hopitaux Universitaires de Strasbourg Strasbourg France; ^6^ Rheumatology Department les Hopitaux Universitaires de Strasbourg Strasbourg France

**Keywords:** LRP5, WNT, OSTEOPOROSIS, BONE, COLLAGEN, FRACTURE

## Abstract

Genetic determinants contribute to osteoporosis and enhance the risk of fracture. Genomewide association studies of unselected population‐based individuals or families have identified polymorphisms in several genes related to low bone density, but not in osteoporotic patients with *Z*‐score < −2.0 SD with fragility fracture(s). The aim of this study was to determine the causal genes of idiopathic osteoporosis in the adulthood. Also, we used next‐generation sequencing of candidate genes in a cohort of 123 young or middle‐aged adults with idiopathic osteoporosis. All patients were included if they had a low bone mineral density (*Z*‐score < −2 SD), a diagnosis before age 55 years (mean ± SD, 48.4 ± 10.6 years; mean ± SD age at first fracture, 30.4 ± 17.4 years) and fracture or not. We found that 11 patients carried rare or novel variants in *COL1A2* (*n* = 4), *PLS3* (*n* = 2), *WNT1* (*n* = 4), or *DKK1* (*n* = 1). We showed a high prevalence of pathogenic variants in *LRP5*: 22 patients (17.8%) had the p.Val667Met variant, including three at the homozygous level and 16 (13%) carrying a novel or very rare variant. Functional analysis revealed that the *LRP5* missense variants resulted in reduced luciferase activity, which indicates reduced activation of canonical WNT signaling. The clinical phenotype of patients carrying causal gene variants was indistinguishable. In conclusion, molecular screening of young osteoporotic adults revealed several variants and could be useful to characterize susceptibility genes for personalizing treatment, in particular for the new anabolic drugs.© 2017 The Authors. *JBMR Plus* is published by Wiley Periodicals, Inc. on behalf of the American Society for Bone and Mineral Research.

## Introduction

Osteoporosis is defined as a skeletal disorder of reduced bone strength leading to increased risk of fracture. Fracture incidence increases with age,^1^, ^2^ so the prevalence is mainly reported in people older than 50 years. Primary osteoporosis, also called idiopathic, in young and middle‐aged patients is rare, and the prevalence is poorly described. Low bone mineral density (BMD) is a major determinant, determined by genetic variation and environmental determinants that contribute to the development of osteoporosis and to enhancing the susceptibility to fracture.^3^

The heritability of BMD is estimated at 60% to 80% in families and twins.^4^, ^5^ Moreover, the heritability of osteoporotic fractures, the final clinical outcome of low BMD, has moderate heritability, 50% to 70%.^6^, ^7^ Genomewide association studies of unselected population‐based individuals have revealed several genes associated with low bone mass and risk of fracture.^8^ Other rare mutations have been reported in young adults with osteoporosis, particularly in extracellular matrix genes or cell factors that regulate bone remodeling. Indeed, mutations in collagen type I (*COL1A1* and *COL1A2*) are responsible for mild to severe osteogenesis imperfecta that can be revealed in adulthood. Heterozygous Wnt family member 1 (*WNT1*) mutations have been found in adults with dominant early‐onset osteoporosis and a homozygous mutation causes severe osteogenesis imperfecta.^9^ Mutations in plastin 3 (*PLS3*),^10^ a gene encoding plastin 3, which is involved in actin bundle formation in the cytoskeleton, have been associated with a X‐chromosomal osteoporosis form.

Among the genes previously identified for low BMD, low‐density lipoprotein (LDL) receptor‐related protein 5 (*LRP5*) is a strong candidate contributing to idiopathic osteoporosis. The encoded LRP5 protein, a member of the LDL receptor superfamily, acts as a coreceptor necessary for Wnt ligands to activate the pathway and regulates bone formation.^11^ *LRP5* was first identified in osteoporosis pseudoglioma syndrome (OPGG, MIM#259770), an exceptional recessive disease characterized by severe osteoporosis revealed in infancy and associated with a congenital blindness. Later, variants were consistently associated with BMD and fracture risk. Two variants of *LRP5—*p.Val667Met (c.2047G>A, population frequency 0% to 5.3%) and p.Ala1330Val (c.4037C>T, population frequency 4.7% to 22%)—had a mild effect on BMD in large non‐selected European cohorts, although it was associated with a significant increased risk of fracture.^3^, ^12^, ^13^ Very few novel heterozygous pathogenic mutations with major effect were identified in children and adolescents with juvenile idiopathic osteoporosis.^14^, ^15^ These studies revealed several variants related to a large spectrum of bone fragility.

Here, we aimed to use next‐generation sequencing (NGS) to screen causal genes in a cohort of young adults with primary osteoporosis and find novel pathogenic variants.

## Patients and Methods

### Patients

Patients were referred to the clinic for management of osteoporosis diagnosed by BMD tests or history of fracture without any associated disease. Enrolment period was from 2010 to 2016. We excluded patients with obvious rare diseases of bone fragility such as osteogenesis imperfecta. Clinical examination showed no dysmorphia and no eye, ear, or vessel abnormalities. Extensive radiological and biochemical investigations excluded secondary causes such as malignant disease, Paget disease, malabsorption, hypogonadism, hemochromatosis, hyperthyroidism, hypercortisolism, vitamin D deficiency, or mastocytosis. We excluded patients taking medications that interfered with bone metabolism. The remaining patients were included in a cohort of young patients with idiopathic osteoporosis; patients were <55 years old at diagnosis and had a BMD *Z*‐score < −2 standard deviation (SD) at the spine or total hip^16^, ^17^ associated or not with osteoporotic fractures. For each patient, the following data were collected: age at diagnosis, number and site of fractures, family history of fracture, and bone marker levels before any treatment. Patients provided informed consent for DNA analysis.

### DNA sequencing and multiplex ligation‐dependent probe amplification technologies

DNA was extracted with use of the QIAsymphony automat according to the manufacturer's protocol (Qiagen, Courtabeuf, France). All DNA was screened by NGS with a panel of five genes reported in adult primary osteoporosis, *LRP5*, *PLS3*, *COL1A1*, *COL1A2*, and *WNT1*, and other potential candidates in osteoporosis, Engrailed Homeobox 1 (*EN1*), Wnt family member 16 (*WNT16*), LDL receptor related protein 6 (*LRP6*) and Dickkopf Wnt signaling pathway inhibitor 1 (*DKK1*), TNF superfamily member 11 (*TNFSF11*), TNF receptor superfamily member 11a (*TNFRSF11A*), TNF superfamily member 11b (*TNFRSF11B*), and vitamin D receptor (*VDR*). The NGS technology was based on the surelectQXT kit (Agilent, Les Ulis, France) for library preparation and the hybrid capture system for sequencing on a Miseq sequencer (Illumina, Paris, France). Fastq files were generated by using Miseqreporter (Illumina), and then aligned with SeqNext (JSI Medical Systems, Ettenheim, Germany) and Surecall (Agilent) software. The design included the coding region of following genes with RefSeq nomenclature: alkaline phosphatase, liver, bone, and kidney (*ALPL* (NM_000478), *COL1A1* (NM_000088), *COL1A2* (NM_000089), *EN1* (NM_001426.3), *LRP5* (NM_002335), *LRP6* (NM_002336.2), *PLS3* (NM_005023), *TNFSF11* (NM_003701), *TNFRSF11A* (NM_001270949), *TNFRSF11B* (NM_002546), *VDR* (NM_001017536), *WNT1* (NM_005430), *WNT16* (NM_057168) for exon regions and intron‐exon boundaries, and *ALPL* (12 exons, NG_008940), *COL1A1* (51 exons, NG_007400), *COL1A2* (52 exons, NG_007405), *EN1* (NG_007123), *LRP5* (23 exons, NG_015835), *LRP6* (NG_016168), *PLS3* (18 exons, NG_012518), *WNT1* (4 exons, NG_033141), *TNFSF11* (8 exons, NG_008990), *TNFRSF11A* (12 exons, NG_008098), *TNFRSF11B* (5 exons, NG_012202), *WNT16* (4 exons, NG_029242), and VDR (NG_008731) for exon numbering and the intronic region.

All variants were confirmed by Sanger technology with Life Technologies products and software on an ABI3130 sequencer (ThermoFischer, Les Ulis, France). Large deletions of *LRP5*, *COL1A1*, and *COL1A2* involved use of multiplex ligation‐dependent probe amplification (MLPA) reagent on the ABI3130 sequencer with coffalyser software (MRC, Amsterdam, Netherland). Variant pathogenicity was evaluated by using the prediction software Polyphen (http://genetics.bwh.harvard.edu/pph2), Mutationtaster (http://www.mutationtaster.org), and SIFT (http://sift.bii.a-star.edu.sg). The known pathogenic mutations were searched in the Human Gene Mutation database (HGMD) or Leiden Open Variation Database (LOVD). All novel variants will be reported in ClinVar database.

### Biochemical markers of bone

Blood samples were collected to assess bone‐remodeling biomarkers. We used the following methods to measure biomarkers: β‐Crosslaps, P1NP, Osteocalcin (Cobas e601 Analyzer; Roche Immunodiagnostics, Meylan, France), bone alkaline phosphatase (Ysis analyzer; IDS Immunodiagnostic, Paris, France), and TRAP5B levels by ELISA kit (IDS Immunodiagnostic).

### Cell culture, transfection, and luciferase reporter assays

The wild‐type construct of the full‐length LRP5 expression plasmid, cloned into the pcDNA3 vector (Invitrogen), was kindly provided by Dr. M. Warman (Harvard Medical School, Boston, MA, USA). The site‐directed mutagenesis involved use of the QuikChange Lightning Multi Site‐Directed Mutagenesis Kit (Agilent). Saos‐2 cells were co‐transfected by using Lipofectamine 2000 (Invitrogen, Paris, France) with the TOPflash‐luc reporter plasmid, pCMV‐Renilla luciferase (Promega, Charbonniere‐les‐bains, France) and an expression‐mutated or control plasmid (expression plasmid without cDNA of LRP5 and expression plasmid with wild‐type cDNA of LRP5). Saos‐2 cells were transfected only with TOPflash‐luc reporter plasmid, pCMV‐Renilla luciferase (a transcription factor activated by Wnt signaling). All experiments were realized in triplicate. Cells were plated at 1.25 × 105 cells/well in 24‐well plates for transfection. Cells were cultured in Wnt3a‐conditioned medium or not for 48 hours before transfection.^18^ The luciferase assay involved the Dual‐Glo Assay system (Promega) according to the manufacturer's instructions. Data were normalized by *Renilla* firefly activity and are presented as a fold change compared to vehicle‐treated cells.

### Statistical analysis

Statistical analysis was conducted in the 112 patients carrying *LRP5* variants or no variant (74 patients in Negative or p.Ala1330Val group, 22 in p.Val667Met group and 16 in Novel or very rare group). Diagnostic tests were performed to detect non‐normality of data and outliers. Bartlett's test was used to test homoscedasticity. Quantitative variables are expressed by mean ± SD. Qualitative variables are expressed by percentages. ANOVA was used to compare multiple groups, followed by a post hoc test in case of a significant effect. The post hoc test consisted of a pairwise comparison by using *t* tests with pooled SD, and *p* value adjustment to control for false discovery rate. Chi‐square test was used to assess the association between genetic groups and gender, and because the theoretical effectives were <5 for the fracture approach, we used Fisher exact tests to study the association with genetic group variables. All tests were two‐sided, and the significance level was 0.05. All statistical analyses involved use of R 3.1.0 (R Core Team, R Foundation for Statistical Computing, Vienna, Austria, 2014; https://www.R-project.org).

## Results

### Characteristics of the cohort

In total, 123 white patients (82 men) fulfilled the criteria for idiopathic osteoporosis. The mean age at the first clinical visit was 48.4 ± 10.6 years, but the first osteoporotic fracture occurred at a mean age of 30.4 ± 17.4 years. The mean *Z*‐score was −2.90 ± 0.90 at the spine and −1.92 ± 0.85 at the hip. Among the 123 patients, 98 experienced at least one fracture, with a high variable number of fractures per patient, as illustrated by a median of three fractures (range, 1 to 14 fractures).

**Figure 1:**
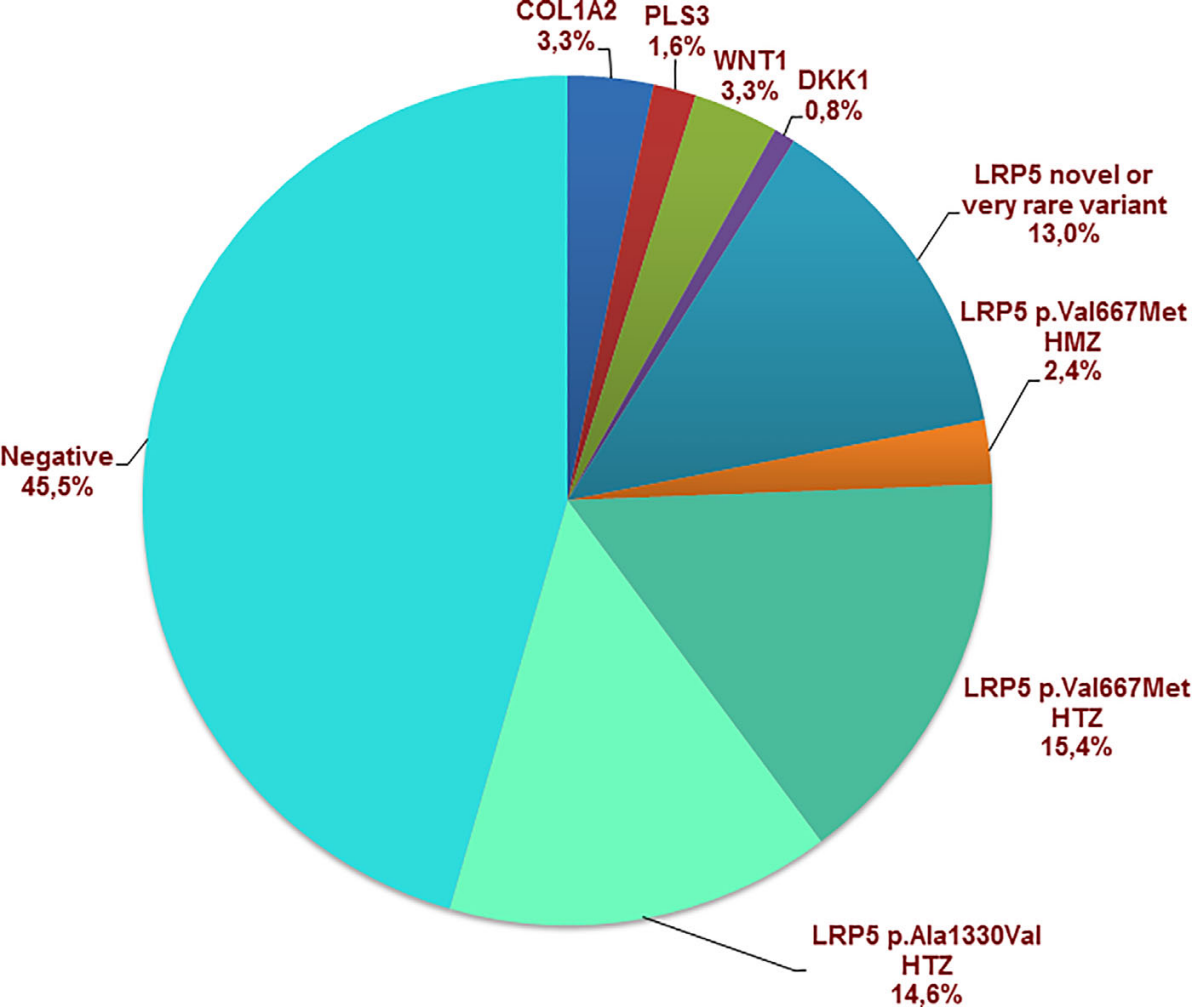
Distribution of gene variants in young patients with idiopathic osteoporosis (*n* = 123). NGS sequencing was used to screen patients with idiopathic osteoporosis (*Z*‐score < −2 SD); 11 patients carried rare variants in COL1A2, PLS3, WNT1, and DKK1. Most patients carried LRP5 common and rare variants and 10 new variants were identified. HTZ = heterozygous; HMZ = homozygous.

NGS sequencing revealed mutations in genes described in several rare diseases associated with bone fragility in 11 patients: *COL1A2* (*n* = 4), *PLS3* (*n* = 2), *WNT1* (*n* = 4), and *DKK1* (*n* = 1) (Fig. 1). In total, 56 patients (35 men) carried *LRP5* variants: 18 with the p.Ala1330Val variant alone (14.5%); 22 with the p.Val667Met variant (17.8%), including three at the homozygous level (1.6%); and 16 carrying a novel or very rare variant (13%). Finally, 56 patients (45.5%) harbored no variants of genes included in the panel (*COL1A1*, *ALPL*, *EN1*, *LRP5*, *LRP6*, *WNT16*, *TNFSF11*, *TNFRSF11A*, and *TNFRSF11B* genes). Clinical and DXA characteristics of the cohort are reported in Table 1.

**Table 1:** Clinical and DXA Characteristics of Idiopathic Osteoporosis Cohort

	COL1A2 *n* = 4 mean ± SD	PLS3 *n* = 2 mean ± SD	WNT1 *n* = 4 mean ± SD	DKK1 *n* = 1 mean ± SD	LRP5 novel or very rare variant *n* = 16 mean ± SD	LRP5 p.Val667Met HMZ *n* = 3 mean ± SD	LRP5 p.Val667Met HTZ *n* = 19 mean ± SD	LRP5 p.Ala1330Val HTZ *n* = 18 mean ± SD	Negative *n* = 56 mean ± SD
Age (yrs)	39.6 ± 10.7	50 ± 4.2	55.5 ± 6.4	47	46.6 ± 11.6	49.7 ± 13.8	44.1 ± 7.5	51.5 ±	49.9 ± 11
Weight (kg)	69.7 ± 18.9	80.5 ± 0.7	63 ± 11.3	85	68.8 ± 12.7	58.2 ± 6.7	64 ± 11.2	69.5 ± 10.1	68 ± 11.9
Height (cm)	170.2 ± 12.7	173 ± 16.3	170 ± 14.1	180	169.7 ± 5.2	166.5 ± 11.3	168.7 ± 7.2	171.1 ± 8.6	170.3 ± 8.7
BMI (kg/m^2^)	23.9 ± 4.8	27.3 ± 5.4	21.7 ± 0.3	26.2	23.7 ± 3.8	20.9 ± 0.5	22.5 ± 3.8	23.7 ± 2.7	23.4 ± 3
BMD L_1_–L_4_ (g/cm^2^)	0.721 ± 0.178	0.818 ± 0.139	0.690 ± 0.04	0.724	0.741 ± 0.113	0.769 ± 0.058	0.786 ± 0.127	0.790 ± 0.105	0.807 ± 0.112
Zscore BMD L_1_–L_4_	–3.7 ± 1.2	–3.1 ± 1.4	–3.1 ± 0.8	–3.1	–3.5 ± 0.8	–3.1 ± 0.7	–3.1 ± 0.9	–2.8 ± 1.0	–2.6 ± 0.8
Tscore BMD L_1_–L_4_	–3.9 ± 1.5	–3.1 ± 1.1	–3.6 ± 0.8	‐	–3.8 ± 0.9	–3.3 ± 0.4	–3.2 ± 1	–3.1 ± 0.9	–3 ± 0.8
BMD total hip (g/cm^2^)	0.836 ± 0.192	0.718 ± 0.005	0.630 ± 0.120	0.867	0.765 ± 0.099	0.802 ± 0.088	0.775 ± 0.107	0.742 ± 0.099	0.760 ± 0.121
*Z*‐score BMD total hip	–1.0 ± 1.1	–2 ± 0	–1.7 ± 0.5	–1	–1.8 ± 0.9	–1.4 ± 0.2	–1.7 ± 0.8	–1.5 ± 0.6	–1.3 ± 0.8
*T*‐score BMD total hip	–1.3 ± 1.3	–2.4 ± 0.1	–2.5 ± 0.6	‐	–2 ± 0.8	–1.9 ± 0.5	–1.7 ± 1.1	–2.1 ± 0.7	–1.9 ± 0.8

HTZ = heterozygous; HMZ = homozygous.

Table 2 summarizes the clinical data for 11 patients and variants in genes previously reported in osteogenesis imperfecta and idiopathic osteoporosis. Four patients showed variants in *COL1A2*. We found two known pathogenic heterozygous mutations p.(Gly193Ser) (HGMD ID CM062556) and p.(Gly247Cys), known at this location in HGMD and in LOVD, in patients 1 and 2 presenting one vertebral fracture and one peripheral fracture associated with markedly low spinal BMD. The known p.(Arg708Gln) variant in *COL1A2* was identified in one woman (patient 4) with fractures from childhood.^19^ The pathogenicity was verified by the SIFT, Mutationtaster, and Polyphen software.

**Table 2:** Description of Variants Except *LRP5*

No	Gender	Gene	Exon	cDNA	Protein	Mutation status	Mutation type	Allele frequency from ExAc	Polyphen (score) pathogenicity 0.5 to 1	BMD spine *Z*‐score	BMD hip *Z*‐score	Number of vertebral fractures (*n*)	Peripheral fractures	Age at first fracture (years)
1	M	COL1A2	12	c.577G>A	p.(Gly193Ser)	HTZ	Missense	HGMD^a^	1	–5.2	–2.3	1	Elbow, tibia	14
2	M	COL1A2	16	c.739G>T	p.(Gly247Cys)	HTZ	Missense	HGMD^a^	1	–5.4	–2.5	1	Ribs, femur	8
3	M	COL1A2	25	c.1412C>T	p.(Pro471Leu)	HTZ	Missense	8.10^–6^ rs72658163	0.6	–3.4	0.2	0	Femur	35
4	F	COL1A2	35	c.2123G>A	p.(Arg708Gln)	HTZ	Missense	0.078% in European population	1	–2.4	–0.9	1	Elbow, wrist	Childhood
5	M	WNT1	1	c.107G>A	p.(Gly36Asp)	HTZ	Missense	–	1	–4.1	–1.6	0	Ribs	40
6	F	WNT1	3	c.436G>C	p.(Gly146Arg)	HTZ	Missense	–	1	–3.6	–3.1	0	Femur, wrist, tibia	10
7	F	WNT1	3	c.401G>T	p.(Gly134Val)	HTZ	Missense	–	1	–4.3	–2.4	1	0	26
8	F	WNT1	3	502G>A	p.(Arg182Trp)	HTZ	Missense	–	1	–3	–1.3	1	Wrist, foot	13
9	M	PLS3	11	1206dup	p.(Val403Argfs*7)	HMZ	Frameshift	–	–	–2.3	–2.5	5	Wrist, femur, humerus	13
10	M	PLS3	18	c.1876G>A	p.(Gly626Arg)	HMZ	Missense	–	1	–3.9	–2.4	1	Metatarsus, clavicle, scapula	18
11	M	DKK1	3	c.359G>T	p.(Arg120Leu)	HTZ	Missense	rs149265042 0.3048%	1	–3	–1	1	0	47

HTZ = heterozygous; HMZ = hemizygous.

^a^ HGMD: human genetic mutation database.

The coding *COL1A2* p.(Pro471Leu) variant of unknown significance (VUS) in patient 3 was not found in the dbSNP database, but was reported with the following allele frequency 8.827e–06 in the Exome Aggregation Consortium database (ExAc) with a potential damaging effect according to the SIFT, MutationTaster, and Polyphen software. This VUS was, however, associated with an unexpected fracture of the femur although femoral BMD was within the normal range in our case. Four novel heterozygous variants of *WNT1* were identified: p.(Gly36Asp), p.(Gly134Arg), p.(Asp168Asn), and p.(Arg182Trp). These variants were not found in any database (ExAc, dbSNP, and Exome Sequencing Project [ESP]), but were considered probably pathogenic according to the prediction software Polyphen‐2, MutationTaster, and SIFT. The clinical expression was variable among the four patients in terms of vertebral or peripheral fractures and age of occurrence of the first fracture, despite low spinal BMD. Patients 9 and 10 harbored novel hemizygous mutations in *PLS3*: p.(Val403Argfs*7) and p.(Gly626Arg), respectively. These two men had vertebral and peripheral fractures with low BMD at both sites. Patient 12 harbored the rare variant, c.359G>T; p.(Arg120Leu) in *DKK1* was previously described^(15)^ and revealed by a vertebral fracture with low BMD at middle age.

### Novel and known variants in *LRP5* gene

We found 112 patients (75 men) carrying *LRP5* variants or no variant: 18 (13 men) with the p.Ala1330Val variant alone; 22 (14 men) with the p.Val667Met variant, including three at the homozygous level; and 16 (8 men) carrying novel or very rare variants (Fig. 1). Because the p.(Ala1330Val) variant has been considered a polymorphism with no detrimental effect on BMD and fractures,^(12,20)^ p.(Ala1330Val) carriers and patients without any *LRP5* variant (*n* = 56) were pooled for further analysis. Table 3 shows the clinical characteristics of patients with the p.Ala1330Val or no *LRP5* variant, those with the rare p.(Val667Met) variant, and those with novel or very rare *LRP5* mutants. Patients were similar in age, height, body mass index, and gender ratio. *Z*‐score BMD at the spine was significantly lower with the p.Val667Met variant or novel/rare variants than without a variant. The three groups did not differ in BMD at the hip. Moreover, the proportion of patients with vertebral fractures was significantly higher with the p.Val667Met variant or novel/very rare variants than a p.(Ala1330Val) or no variant.

**Table 3:** Clinical and Density Parameters in Patients With *LRP5* Variants

	Negative or p.Ala1330Val *n* = 74 mean ± SD	p.Val667Met *n* = 22mean ± SD	Novel or very rare *n* = 16 mean ± SD	*p*‐value
Clinical parameters				
Age (yrs)	50.3 ± 10.8	44.9 ± 8.4	46.6 ± 11.6	0.08
Weight (kg)	68.4 ± 11.4	63.1 ± 10.8	68.8 ± 12.7	0.18
Height (cm)	170.5 ± 8.6	168.4 ± 7.6	169.7 ± 52	0.58
BMI (kg/m^2^)	23.4 ± 2.9	22.3 ± 3.6	23.7 ± 3.8	0.30
Women (*n* (%))	21 (28%)	8 (36%)	8 (50%)	0.23
With personal history of fractures (*n* (%))	51 (69%)	20 (91%)	15 (100%)	<10^–1^ ^a^
With vertebral fractures (*n*, (%))^b^	27 (36%)	11 (50%)	12 (75%)	<10^–1^ ^a^
With only peripheral fractures (*n*, (%))	24 (32%)	9 (41%)	4 (25%)	
Areal densitometry				
Areal BMD spine L_1_–L_4_ (g/cm^2^)	0.803 ± 0.110	0.784 ± 0.119	0.741 ± 0.113	0.16
*Z*‐score spine L_1_–L_4_ ^c^	–2.63 ± 0.84	–3.12 ± 0.83	–3.48 ± 0.83	<10^–1^
*T*‐score spine L_1_–L_4_ ^c^	–3.01 ± 0.81	–3.22 ± 0.91	–3.81 ± 0.85	<10^–1^
Areal BMD total hip L_1_–L_4_ (g/cm^2^)	0.755 ± 0.115	0.779 ± 0.103	0.765 ± 0.099	0.68
*Z*‐score total hip L_1_–L_4_	–1.37 ± 0.78	–1.69 ± 0.71	–1.85 ± 0.87	0.12
*T*‐score total hip L_1_–L_4_	–1.98 ± 0.79	–1.74 ± 1.01	–1.97 ± 0.75	0.49

^a^ Fisher's exact test.

^b^ Included vertebral fractures associated with peripheral fractures.

^c^ Pairwise comparisons using *t* tests with pooled SD, *p* value adjustment method: fdr. *Z*‐score spine L1–L4: *p‐value*<10^−1^ between Without variant or p.Val1330 and Exceptional variants; *p‐value*<0.05 between Without variant or p.Val1330 and p.Va1667Met; *p‐value* = 0.26 between p.Val667Met and Exceptional variants. *T*‐score spine L1–L4: *p*‐*value*<10–1 between Without variant or p.Val1330 and Exceptional variants; *p‐value* = 0.31 between Without variant or p.Va11330 and p.Val667Met; *p‐value*<0.05 between p.Val667Met and Exceptional variants.

Biochemical markers were measured in a subgroup of patients before the initiation of treatment. The three groups did not differ in circulating levels of bone alkaline phosphatase, P1NP, osteocalcin, or tartrate‐resistant acid phosphatase. Only serum β‐crosslaps levels were higher in with p.(Val667Met) (483.6 ± 176 pg/mL) or novel/very rare variants (570.6 ± 205.3 pg/mL) than a p.(Ala1330Val) or no variant (385.4 ± 210 pg/mL, *p* < 0.05).

In this cohort, 16 patients harbored heterozygous novel or very rare *LRP5* mutations and three patients harbored homozygous rare LRP5. The clinical and genetic characteristics are in Table 4. The following variants were not found in the databases: p.(Lys265Gln), p.(Cys336Gly), p.(Met473Thr), c.1413–2A>G, p.(Lys772Argfs*26), p.(Ala1418Profs), and p.(Glu1597*). Some variants were reported as rare in a population according to ExAc: p.(Asp587Asn), p.(Trp560Cys), p.(Glu829Gln) p.(Arg1188Gln), p.(Ala1196Thr), and p.(Asp1288Gly). Except for p.(Ala1196Thr), the missense variants were classified as probably pathogenic, with high scores, according to the prediction software Polyphen‐2, MutationTaster, and SIFT (Table 4). The clinical phenotype varied in patients with *LRP5* mutations but was associated with vertebral fractures in 13 of 19 patients. In four patients, the novel/very rare variant was in combination with the p.(Val667Met) variant. The combination was often associated with a very low spinal BMD and high number of vertebral fractures. Patient 7 is a 25‐year‐old woman with the p.(Asp587Asn) *LRP5* mutation associated with the p.(Arg120Leu) variant in *DKK1* at a heterozygous level. This situation was associated with a severe phenotype, as shown by a very low BMD and axial and peripheral fractures. The patient's brother harbored only the *DKK1* variant, with low BMD, and her father presented only the *LRP5* variant, with low BMD. Hence, the phenotype may be more severe in the presence of two variants.

**Table 4:** Description of *LRP5* Variants

No	Gender	Exon/ Intron	cDNA	Protein	Variant status	Mutation type	Association with c.1999G>A, p.Val667Met variant	Allele frequency from ExAc	Polyphen (score) pathogenicity 0.5 to 1	BMD spine *Z*‐score	BMD hip *Z*‐score	Number of vertebral fractures (*n*)	Peripheral fractures	Age at first fracture (yrs)
1	M	E4	c.793A<C	p.(Lys265Gln)	HTZ	Missense	No	–	1	–2.5	–0.1	1	Wrist	10
2	F	E5	c.1006T>G	p.(Cys336Gly)	HTZ	Missense	No	–	0.881	–5.4	–2.5	1	Ribs	40
3	F	I7	c.1413–2A>G	–	HTZ	STOP	No	–	–	–3.6	–1.3	0	0	14
4	M	I7	c.1413–2A>G	–	HTZ	STOP	Yes	–	–	–4.1	–2.1	3	Wrist, ribs	6
5	M	E7	c.1418T>C	p.(Met473Thr)	HTZ	Missense	Yes	–	0.935	–4.2	–1.9	3	Wrist, clavicle, humerus, ribs	10
6	F	E8	c.1680G>T	p.(Trp560Cys)	HTZ	Missense	No	4.945e–05	0.994	–3.1	–1	2	Ribs	26
7	F	E8	c.1759G>A	p.(Asp587Asn)	HTZ	Missense	No	–	0.844	–4.5	–3.3	2	Humerus	10
8	M	E9	c.1999G>A	p.(Val667Met)	HMZ	Missense	–	0.03767	0.993	–3.9	–1.6	1	0	34
9	M	E9	c.1999G>A	p.(Val667Met)	HMZ	Missense	–	0.03767	0.993	–3.6	–1.6	0	0	–
10	M	E9	c.1999G>A	p.(Val667Met)	HMZ	Missense	–	0.03767	0.993	–2.6	–1.3	3	Fibula	50
11	F	E10	c.2313delC	p.(Lys772Argfs*26)	HTZ	Frameshift	No	–	–	–4.4	–2	1	Wrist	10
12	M	E11	c.2485G>A	p.(Glu829Lys)	HTZ	Missense	No	–	0.992	–4.1	–1.1	0	Calcaneus, ribs	46
13	M	E16	c.3563G>A	p.(Arg1188Gln)	HTZ	Missense	No	8.278e–06	0.987	–4.1	–1.6	0	Ribs	13
14	M	E16	c.3586G>A	p.(Ala1196Thr)	HTZ	Missense	Yes	3.322e–05	0.606	–3.8	–1.8	2	Metacarpus	22
15	M	E18	c.3863A>G	p.(Asp1288Gly)	HTZ	Missense	No	1.668e–05	1	–3.5	–1.8	5	Wrist	13
16	M	E20	c.4252delG	p.(Ala1418Pro*21)	HTZ	Frameshift	Yes	–	–	–3.0	–2.0	0	Knee	58
17	M	E23	c.4789G>T	p.(Glu1597*)	HTZ	STOP	No	–	–	–2.4	–2.6	1	0	47
18	F	E18	c.3883T>C	p.(Cys1295Arg)	HTZ	Missense	No	–	–	–3.2	–1.4	1	Tibia	11
19	F	E19	c.4105_4106delAT	p.(Met1369Valfs*2)	HTZ	Frameshift	No	–	–	–3.6	–2.0	0	Ischiopubic branch	48

HTZ = heterozygous; HMZ = homozygous.

### Functional studies of novel *LRP5* variants

**Figure 2:**
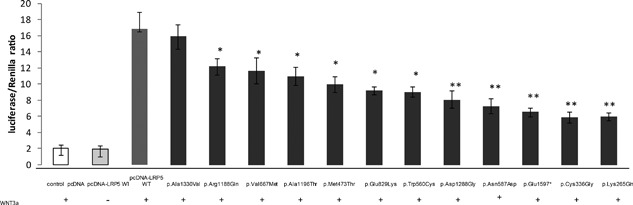
LRP5 variants reduce activation of canonical Wnt signaling. Canonical Wnt signaling was induced by Wnt3a after WT or variant transfection in Saos2 cells. Wnt canonical pathway activity was analyzed by firefly luciferase activity and normalized to renilla luciferase activity. *p < 0.05, **p < 0.01 compared to WT. WT = wild‐type.

To confirm the potential impact of novel or very rare exonic variants in *LRP5* in bone, we performed site‐directed mutagenesis in SaoS2 human osteosarcoma cell lines. Wild‐type (WT) and mutant LRP5 proteins were expressed independently along with a luciferase reporter construct under control of a promoter activated by lymphoid enhancer binding factor 1 (LEF1) (Fig. 2). Cells were cultured in the presence of Wnt3a‐enriched medium for 48 hours. After transfection, *LRP5* missense variants resulted in reduced luciferase activity, which indicates reduced activation of canonical WNT signaling and confirms the pathogenicity of the missense mutation.

## Discussion

We report the clinical and molecular characteristics of a large cohort of patients with idiopathic osteoporosis. With an in‐depth phenotype analysis, we show that this disease form involves a large spectrum of genes related to bone fragility. The contribution of target sequencing to identify a risk factor in young adults revealed mutations in several causal genes—*COL1A2*, *WNT1*, *PLS3*, and *DKK1*—reported in patients with low BMD or fractures. Furthermore, our study provides evidence of variable clinical severity in bone diseases. In four patients harboring *COL1A2* mutations, the phenotype of osteoporosis was indistinguishable from mild osteogenesis imperfecta in young and middle‐aged adults. Moreover, the occurrence of fracture at the mid‐age and the absence of any clinical sign of ear or eye alterations were not in favor of the diagnosis of osteogenesis imperfecta. Indeed, there are no sharp criteria able to discriminate between the two forms. As expected, two mutations in *COL1A2* affect a glycine residue. Indeed, the Gly residues changed by Ser and Cys residues were reported as a mild phenotype of osteogenesis imperfecta,^21^ and the p.Gly193Ser mutation was previously described in osteogenesis imperfecta type IV.^22^ These two patients have extremely low spinal and femoral BMD, with occurrence of the first fracture at a young age. The p.Pro471Leu variant was diagnosed after a femoral fracture revealing low spinal BMD, but occurred with normal hip BMD, which suggests reduced bone strength independent of bone density. Indeed, proline residues along the helical regions of both chains required hydroxylation by prolyl4‐hydroxylase 1, and the subsequent substitution of Pro could modify the structure of the collagen chains and induce lower bone resistance.^23^, ^24^, ^25^ Finally, the very rare p.Arg708Gln variant in *COL1A2* was associated with a severe osteoporosis phenotype previously reported in an older patient.^19^ A striking finding is that these patients were *COL1A2* carriers with variable severity in bone phenotype, but none carried *COL1A1* variants. Indeed, the phenotype was restricted to bone, and no patient had blue sclera or deafness that could have suggested a form of osteogenesis imperfecta.

Our study shows the large spectrum of bone fragility in young adults with *COL1A2* mutations that could be revealed at a young or middle age in the absence of other clinical signs. Therefore, this form of bone fragility is at the border of idiopathic osteoporosis and osteogenesis imperfecta, the difference being that osteogenesis imperfecta with *COL1A1* mutations also affects eyes and ears rather than just bone. With this series, we also confirmed a low frequency of *PLS3* and *WNT1* mutations in patients showing the first fractures in infancy, with a bone phenotype similar to those with *COL1A2*.^10^, ^26^, ^27^ The characterization of a genetic background in idiopathic osteoporosis was facilitated by the use of NGS sequencing, which would not have been possible by Sanger sequencing targeting collagen I only.

Another key finding is the high prevalence of variants in *LRP5*, a main co‐receptor that triggers the Wnt pathway and osteoblast activity. Several studies and a meta‐analysis failed to show an association between fractures or low BMD and the p.Ala1330Val polymorphism, so this polymorphism is not considered a risk factor for low BMD or fracture.^12^, ^20^ The frequency of 14.5% of the variation in our cohort is lower than the 22% previously reported in white population‐based cohorts with ExAc. Moreover, the frequency of the heterozygous p.Val667Met variant in *LRP5* reached 15% in our selected population but was reported as 5.36% in Europe in databases such as dbSNP, ExAc, and ESP. The unexpected high frequency at the homozygous level (frequency according to ExAc, 0% to 0.15%) might be explained by the selected population being BMD‐based and fracture‐based rather than population‐based. Indeed, three patients harbored the homozygous p.Val667Met variant, although osteoporosis and fractures occurred in adulthood without eye involvement, as described in osteoporosis pseudoglioma syndrome.^28^ The absence of a full phenotype despite the homozygous variant suggests that this variant is not a full loss of function in osteoporosis pseudoglioma syndrome phenotype. Here, heterozygous/homozygous p.Val667Met and novel variants in *LRP5* were found associated with a significantly lower *Z*‐score BMD, which increases the risk of fractures. Remarkably, the occurrence of vertebral fractures is significantly higher in the presence of *LRP5* variants than in the absence of pathogenic mutation. Therefore, severe and symptomatic osteoporosis could be used to identify pathogenic variants in young adults. The p.Val667Met variant has a pathogenic effect similar to the novel or very rare variant in our cohort, which should be further investigated in terms of bone fragility. This approach would also be of interest for identifying patients at high risk of fracture in terms of further care. Thus, such variants could be considered as susceptibility factor and might justify a first‐line treatment with anabolic treatment.

The novel and very rare mutations identified in *LRP5*, including missense, splicing, frameshift, and nonsense mutations, are associated with low BMD. Also, the functional study of missense and nonsense mutations confirmed their pathogenicity by showing reduced Wnt signaling activity induced by Wnt3a. This is in line with previous missense Lpr5 mutations reported with functional effects.^29^ Surprisingly, the very rare *LRP5* variant p.Trp560Cys was also recently reported in autosomal‐dominant polycystic kidney disease.^30^ In addition, the highly conserved amino acid residue Arg1188 was found associated with isolated polycystic liver disease, a common extrarenal manifestation in autosomal‐dominant polycystic kidney disease.^31^ No bone phenotype was available in the case report of p.Arg1188Trp mutation in polycystic liver disease, while our patient with the heterozygous p.Arg1188Gln mutation did not have polycystic kidney or liver disease. Indeed, the p.Trp560Cys mutation presents a distinct clinical presentation. Further studies are required to elucidate how unique mutations in *LRP5* might be responsible for a wide spectrum of complex diseases with tissue‐specificity. The heterozygous p.Arg120Leu variant in *DKK1*, an inhibitor of the Wnt pathway, suggests its likely contribution to bone fragility.^32^ The mutated inhibitor of Wnt has a higher affinity for *LRP5*, although this finding needs to be confirmed. The adult patient harboring only a *DKK1* variant had a mild phenotype, in contrast to the female patient with severe juvenile osteoporosis with a combination of *LRP5* and *DKK1* variants, so the combination of two variants in the same pathway may accentuate the phenotype.

Despite the identification of several variants in causal genes, we failed to find any variant that could explain the low BMD in 45.5% of patients. The NGS sequencing panel included the WNT16 ligand, uncovered by some genomewide association studies. However, no mutation was identified in the coding region of *WNT16* gene in our cohort despite the role of the WNT16 ligand in bone remodeling affecting both osteoclastogenesis and osteoblastogenesis. This finding would indicate that *WNT16* mutation is rare or would promote a specific phenotype distinct from the idiopathic osteoporosis. Also, *EN1*, coding for Homeobox protein engrailed 1, was found recently by whole genome sequencing. Intronic polymorphisms in *EN1* were associated with low BMD and high risk of fracture, but we found no patient with such mutation.

In conclusion, NGS target sequencing allowed for characterizing the genetic background in young osteoporotic patients, showing that several genes might be associated with a similar bone phenotype. *LRP5* appears to be a major gene that could explain in part the low BMD and fractures in a selected population, which suggests that the failure of the Wnt pathway contributes to idiopathic osteoporosis. Molecular screening in young adults could be useful to identify susceptibility genes for fractures in terms of personalized treatment, in particular for future anabolic therapies.

## Disclosures

Martine Cohen‐Solal received lecture fees from Amgen. All the other authors have no conflict of interest.
